# Postoperative Magnetic Resonance Imaging following Arthroscopic Primary Anterior Cruciate Ligament Repair

**DOI:** 10.1155/2019/5940195

**Published:** 2019-03-26

**Authors:** Jelle P. van der List, Douglas N. Mintz, Gregory S. DiFelice

**Affiliations:** ^1^Orthopaedic Trauma Service, Department of Orthopaedic Surgery, Hospital for Special Surgery, NewYork-Presbyterian, Weill Medical College of Cornell University, New York, NY, USA; ^2^Spaarne Gasthuis Hospital, Department of Orthopaedic Surgery, Hoofddorp, Netherlands; ^3^Amsterdam UMC, University of Amsterdam, Department of Orthopaedic Surgery, Amsterdam, Netherlands; ^4^Department of Radiology, Hospital for Special Surgery, NewYork-Presbyterian, Weill Medical College of Cornell University, New York, NY, USA

## Abstract

**Introduction:**

Recently, there has been a resurgence of interest in arthroscopic primary anterior cruciate ligament (ACL) repair. To date, no studies have assessed the role of postoperative magnetic resonance imaging (MRI) on the status and maturation of the repaired ligament. The goal of this study was therefore to assess (I) the accuracy of MRI on rerupture of the repaired ligament and (II) the maturation of the repaired ACL.

**Methods:**

All postoperative MRIs of patients that underwent arthroscopic primary ACL repair were included. A musculoskeletal radiologist, blinded for MRI indication, surgery-MRI time interval, and clinical stability, retrospectively assessed the ligament continuity and graded ligament maturation as hypointense (similar to intact PCL), isointense (>50% similar to PCL), or hyperintense (<50% similar to PCL).

**Results:**

Thirty-seven MRIs were included from 36 patients. Mean age was 30 years (range: 14–57 years), and mean surgery-MRI interval was 1.5 years (range: 0.1–4.9 years). The radiologist recognized 6 out of 8 reruptures and 26 out of 29 intact ligaments (sensitivity 75%, specificity 90%, and accuracy 86%). Ligaments in the first year were more often hyperintense than after one year (60% vs. 11%,* p*=0.02), most often isointense (60%) between one and two years, and more often hypointense after two years than before two years (56% vs. 10%,* p*=0.03).

**Conclusion:**

Postoperative MRI was found to accurately predict the rerupture of the primarily repaired ACL. Furthermore, it can be expected that the repaired ligament is hyperintense within the first year, while the signal becomes similar to the intact PCL after two years.

## 1. Introduction

Approximately 120 years ago, Mayo Robson was the first to surgically treat an anterior cruciate ligament (ACL) injury using open primary repair [1]. Over the following decades, Ivar Palmar and Don O'Donoghue further popularized this treatment and open primary repair became an important surgical treatment for ACL injuries in the 1970s and 1980s [1–6]. Although the short-term outcomes were promising [7–9], Feagin and Curl [10] and others [11–16] noted that the outcomes deteriorated at longer-term follow-up and as a result, the technique was abandoned and ACL reconstruction became the gold standard for all ACL injuries [6].

Several may have negatively influenced the early outcomes of primary repair [6]. Firstly, primary repair was historically used for patients with all tear types, while in hindsight better outcomes were found in patients with proximal tear types [16–19]. Secondly, primary repair was historically performed with an invasive open procedure (arthrotomy), while minimally invasive surgery (arthroscopy) decreases morbidity of the procedure [20]. Finally, patients were historically immobilized for four to six weeks postoperatively, while regaining early range of motion is now known to improve outcomes [21–23].

By applying several modern developments, such as magnetic resonance imaging (MRI) for appropriate patient selection [24], arthroscopy for minimal invasive surgery [25], and early rehabilitation to prevent stiffness [23], better outcomes of primary repair can be expected. Indeed, DiFelice et al. showed excellent outcomes of primary repair of proximal ACL tears using minimally-invasive arthroscopy and focusing on early rehabilitation [26]. More recently, others have confirmed these promising outcomes [27–38]. In the reconstruction literature, many studies have assessed the role of postoperative MRI on graft maturation [39–42], but studies assessing the maturation of the repaired ACL following arthroscopic primary repair are currently lacking.

The goal of this study was therefore to assess the role of postoperative MRI following arthroscopic primary repair of proximal ACL tears. We hypothesized that postoperative MRI can be used to accurately assess (I) rerupture of the repaired ACL and (II) maturation of the repaired ACL.

## 2. Methods

### 2.1. Patient Selection

After Institutional Regional Board approval was obtained, a retrospective search was performed in the database of the senior author (GSD) for patients treated who had a postoperative MRI following arthroscopic primary repair between January 2008 and August 2018. The senior author has performed arthroscopic primary ACL repair in patients with proximal avulsion type tears using the pullout suture technique, suture anchor technique, or suture anchor technique with internal brace that have been previously described [25, 43, 44]. Out of 154 patients treated with arthroscopic primary repair, 37 MRIs of 36 patients could be identified.

### 2.2. MRI Grading

MRIs were performed at different institutions and therefore differed in quality and scanning details. However, all MRIs were minimum 1.5T scans and consisted of sagittal, coronal, and axial views. An experienced musculoskeletal radiologist reviewed all MRIs while blinded for the indication for the MRI, surgery-MRI time interval, and the clinical situation (i.e., stable or unstable knee). Using the sagittal, coronal, and axial views of both T1 and T2 sequences, the radiologist first graded the ligament in all patients as continuous or not continuous (reruptured). No additional sequences were used for this study to have similar MRI images for all patients. The maturation of the ligament was then graded similar to graft maturation in the ACL reconstruction literature [39–42, 45]: hypointense if the intensity of the repaired ligament was similar to the posterior cruciate ligament (PCL) and lower than posterior muscles (i.e., gastrocnemius muscle and semimembranosus), isointense if more than 50% of the ligament had the same intensity as the PCL and intensity was similar to posterior muscles, or hyperintense if less than 50% of the ligament had the same intensity as the PCL and more intensity than the posterior muscles. No computational software was used for this grading and the grading was performed on both T1 and T2 images and mainly based on the T1 images. An overview of the definitions of ligament intensity is provided in Table 1.

### 2.3. Data Collection

Data collected from the patients were data on the operative procedure, gender, age, BMI, time from surgery to MRI, reason for MRI, and clinical examination before and after MRI. Clinical examination was considered unstable if there was a minimum 2+ Lachman or 2+ pivot shift testing. All 37 MRIs were used to assess the accuracy of ACL rerupture, and all clinically stable and continuous ligaments were used to assess maturation in three time intervals based on ACL reconstruction literature [39–42, 45, 46]: MRI within one year of surgery, between one and two years of surgery, and after two years of surgery.

### 2.4. Statistical Analysis

Statistical analysis was performed using SPSS Version 24 (SPSS Inc., Armonk, NY, USA). Sensitivity, specificity, positive predictive value, negative predictive value, and accuracy were calculated using two by two tables. Fisher's exact test was used to compare the intensity of the ligament at different postoperative intervals. All tests were two-sided and differences with* p* < 0.05 were considered statistically significant.

## 3. Results

### 3.1. Baseline Characteristics

Thirty-seven MRIs from 36 patients were included in this study. Mean age of patients was 30 years (range: 14–57), 20 patients were male (57%), and mean time from operation to MRI was 1.5 years (range: 0.1–4.9 years). Eight MRIs (22%) were obtained in patients who suffered new trauma and had clinically unstable knees on clinical examination. Six of these eight unstable patients (75%) underwent arthroscopy in which the rerupture was confirmed. Of the 29 stable knees (78%), 10 (34%) were obtained within one year of surgery, 10 (34%) between one and two years of surgery, and 9 (30%) more than two years after surgery. Reasons for MRI were knee pain without trauma (n = 12; 32%), trauma with high suspicion for ACL re-injury (n = 9; 24%), evaluation of ligament healing (n = 8; 22%), trauma with low suspicion for ACL re-injury (n = 4; 11%), superficial tenderness over tibial internal brace suture anchor (n = 2; 5%), and evaluation of meniscal (root) repair (n=2; 5%) (Table 2).

### 3.2. Accuracy Rerupture of Repaired ACL

**Figure 1:**
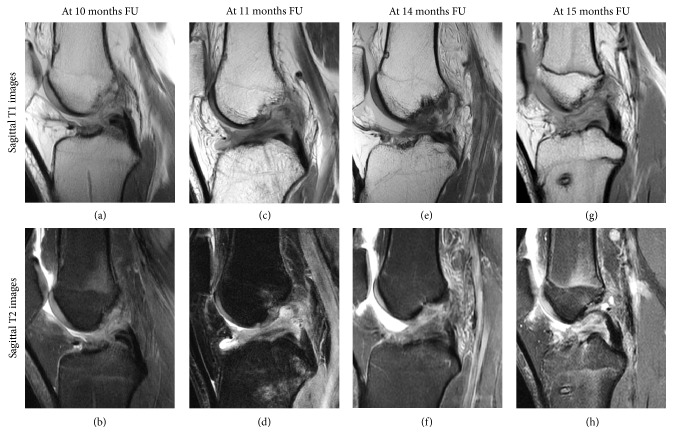
Sagittal T1 (upper row) and T2 (lower row) MR images of four patients are shown with a rerupture of the repaired ligament.

The radiologist graded 6 out of 8 unstable knees as not continuous (Figure 1) and graded 26 out of 29 stable knees as continuous. This corresponded to a sensitivity of 75%, specificity of 90%, positive predictive value of 67%, negative predictive value of 93%, and accuracy of 86% (Table 3).

### 3.3. Maturation of Repaired ACL

**Figure 2:**
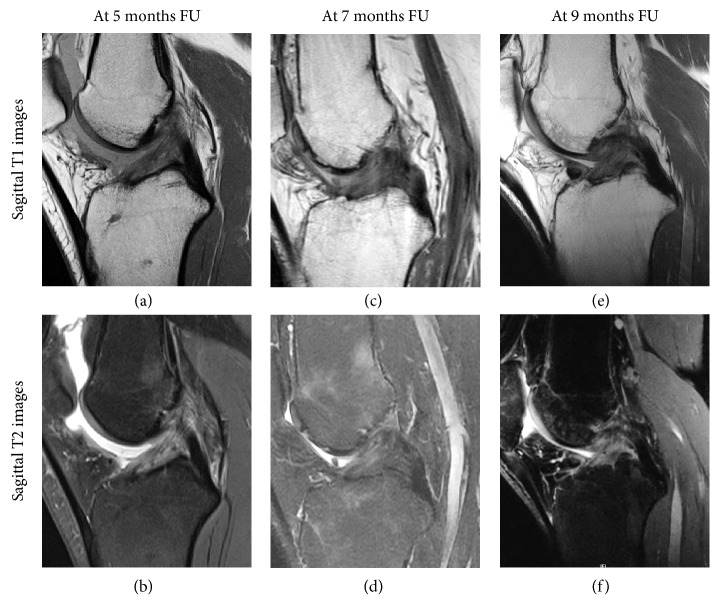
Sagittal T1 (upper row) and T2 (lower row) MR images of three patients are shown within one year after surgery. The ligament in most of these patients is hyperintense as the ligament has more intensity than the PCL and the posterior muscles.

**Figure 3:**
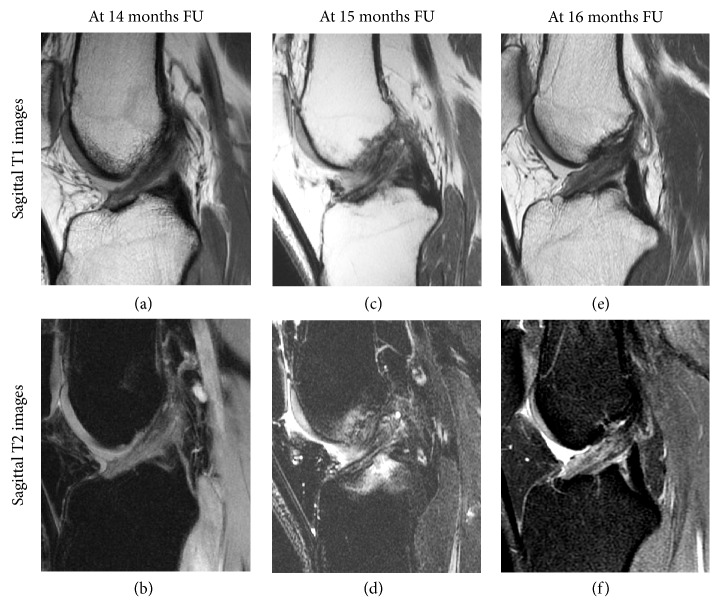
Sagittal T1 (upper row) and T2 (lower row) MR images of three patients are shown between one and two years after surgery. At this time interval, the ligament is often isointense as the ligament has more intensity than the PCL and similar intensity to the posterior muscles.

**Figure 4:**
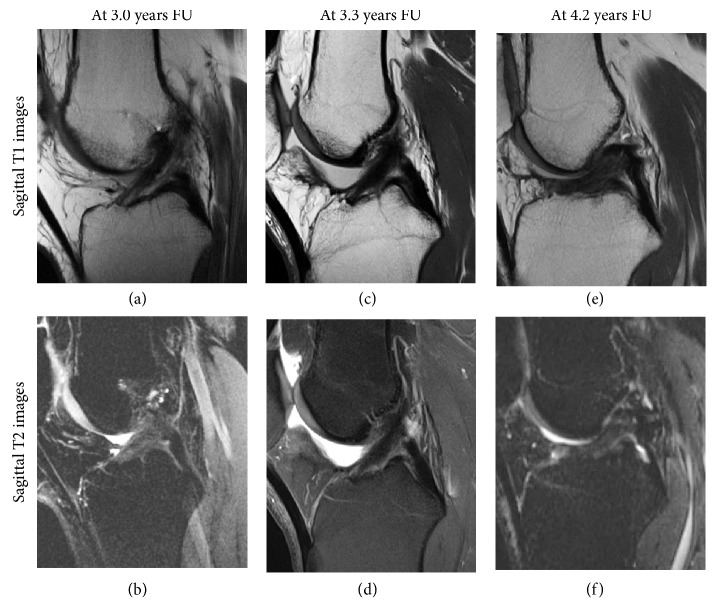
Sagittal T1 (upper row) and T2 (lower row) MR images of three patients are shown more than two years after surgery. The ligament is continuous and the intensity is similar to the PCL and lower than the posterior muscles.

**Figure 5:**
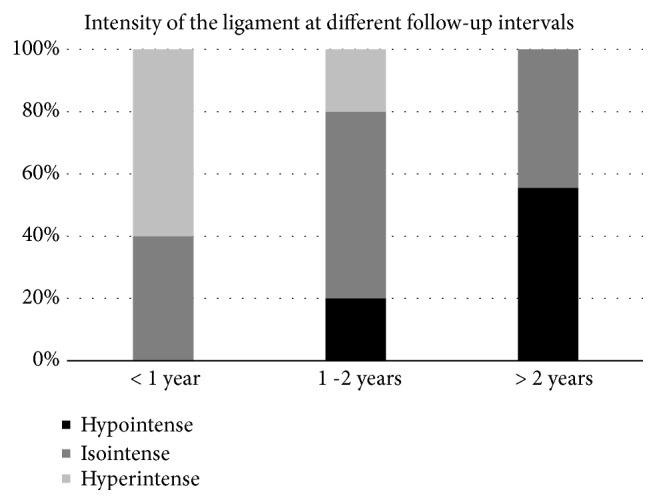
Graph displays the distribution of hypointense (black), isointense (dark gray), and hyperintense (light gray) at different follow-up intervals of the patients that had a functioning ACL (no rerupture).

**Figure 6:**
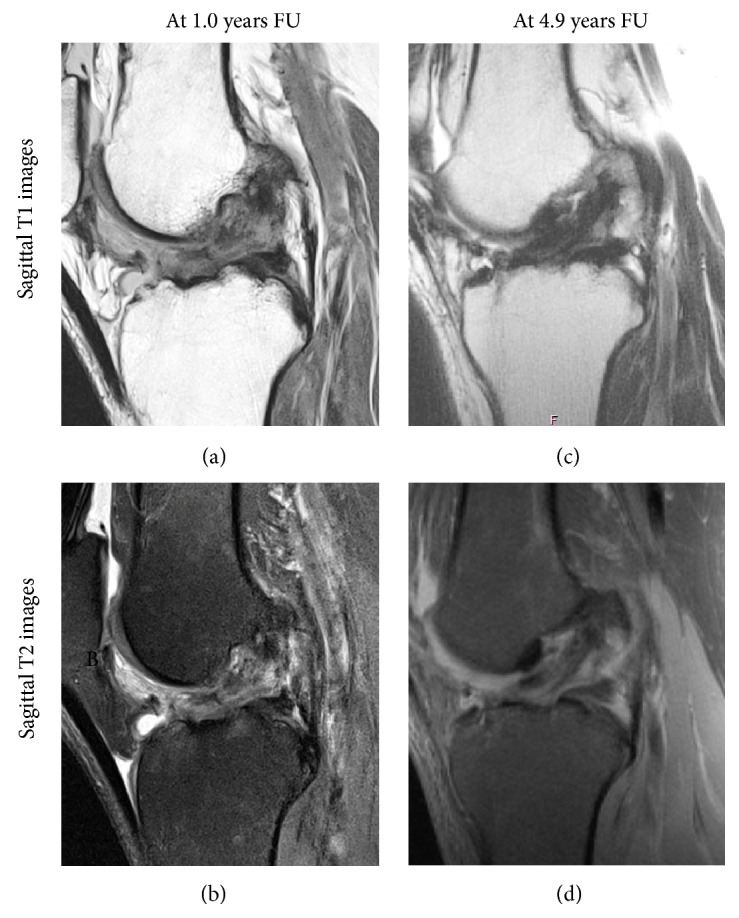
Sagittal T1 (upper row) and T2 (lower row) MR images are shown of the same patient at one and five years of follow-up. At one-year follow-up, the ligament is hyperintense and even appears to have a partial rupture of the ligament. This patient was clinically stable, and no intervention was undertaken. At five-year follow-up, the ligament on MRI was continuous and hypointense.

Within one year of surgery, ligaments were hyperintense (60%) or isointense (40%) and were more often hyperintense than after one year (60% vs. 11%, respectively,* p* = 0.02) (examples in Figure 2). Between one and two years postoperatively, majority of ligaments were isointense (60%) (examples in Figure 3). Minimum two years postoperatively, ligaments were hypointense (56%) or isointense (44%) and were more often hypointense than on MRIs within two years of surgery (56% vs. 10%, respectively,* p* = 0.03) (examples in Figure 4). Overview of the distribution of ligament intensity can be seen in Figure 5, and an early and a late MRI of the same patient can be seen in Figure 6.

## 4. Discussion

The goal of this study was to assess the role of postoperative MRI on the (I) accuracy of ACL rerupture and (II) ligament maturation following arthroscopic primary ACL repair. It was noted that postoperative MRI can accurately assess rerupture of the repaired ligament. Furthermore, it was noted that ligaments were generally hyperintense within one year of surgery, isointense between one and two years, and hypointense after more than two years.

This is the first study assessing the role of postoperative MRI following arthroscopic primary ACL repair and assessing maturation of the repaired ligament. It is therefore only possible to compare these findings with the reconstruction literature or experimental studies on primary repair. Howell et al. were one of the first to use MRI to assess graft maturation following hamstring autograft reconstruction and found that ligament intensity changed significantly during the first year after surgery [40]. More recently, Ntoulia et al. performed a prospective study in which they obtained MRIs at 3 days, 6 months, 12 months, and 24 months following autograft bone-patellar tendon-bone ACL reconstruction [46]. Interestingly, the patients underwent MRI before and after intravenous gadolinium contrast, which enabled them to look at the healing and remodeling of the graft. They noted similar findings as in our current study with significantly increased signal intensity in the intra-articular graft at 6 and 12 months and more homogeneous low signal at 24 months. With the gadolinium scans, they noted that the increased signal intensity was caused by significant revascularization. Extrapolating these findings, it is possible that the hyperintense and isointense repaired ligaments in our study were also caused by healing and remodeling. This would indicate that the time to complete remodeling could take up to two years after arthroscopic primary ACL repair.

Although no clinical studies have assessed the postoperative MRI features following arthroscopic primary repair, the group of Murray has performed many experimental studies on primary repair with a biological scaffold [47, 48]. Biercevicz et al. performed primary repair with biological scaffold in Yucatan minipigs and performed MRI at 15 and 52 weeks of follow-up [47]. Similar to the findings of our study, they noted that the repaired ligaments were more often hypointense at longer follow-up, indicating that the ligament is healing and remodeling. In a first pilot study with ten human patients, Murray et al. assessed the outcomes at three months of follow-up and also obtained MRIs at this interval [49]. Since they did not grade the intensity of the repaired ligament and only displayed sagittal proton density images in their paper, it is difficult to compare their findings to our study. It can, however, be seen that the repaired ligaments in their study were grossly similar to the MRIs within one year of surgery in this current study (i.e., mostly hyperintense).

After the disappointing results of open primary repair in the historical literature, there has been a general consensus that primary repair of the ACL does not work, because the intra-articular synovial environment prevents clot formation and ligament healing [6, 50, 51]. When critically reviewing the literature, however, it seems proximal tears can heal when the ligament has sufficient length to be reapproximated to the femoral wall [17, 19, 52]. In our series of postoperative MRIs, the 29 patients without traumatic rerupture indeed had ligament continuity to the femoral insertion site and clinical stability. This finding, along several histological and clinical studies showing that the healing potential of the proximal ACL is similar to the MCL [52–56], indicates that primary ACL repair can result in healing of proximal tears and is therefore a good treatment for this select group of patients with proximal tears [26].

In this study, sensitivity of 75%, specificity of 90%, and accuracy of 86% were found for rerupture of the repaired ligament. Although it is not possible to compare these findings with other studies assessing this following primary repair, several studies have assessed these MRI characteristics following ACL reconstruction [57–59]. These studies found a sensitivity of 60–100%, accuracy of 85–87%, and specificity of 86–100% for a ruptured graft [57–59]. The MRI characteristics in our study were similar to the MRI characteristics in these studies, which can be expected as there was a clear difference in continuity between stable and unstable knees (Figure 1 versus Figures 2 and 3). In three patients, the radiologists graded a clinically stable ligament as not continuous, which can likely be explained by the early healing and fluid in the proximal part of the ligament that can be seen as a (high-grade) tear in the proximal part. It is therefore important to compare these findings with the clinical stability and that the radiologist is aware of the fact that there is a healing ligament rather than a reconstructed graft.

Limitations are present in this study. First of all, this is a retrospective study in which MRIs were made for several reasons and this was not a prospective study in which the same knees were followed at several time points. Nonetheless, we believe that the findings and MRI examples in this study are valuable for surgeons who perform arthroscopic primary ACL repair and obtain postoperative MRIs. Secondly, the quality of MRIs in this study varied as studies were performed at different institutions. Despite this lack of standardization, we believe this increases the external validity of the findings. Furthermore, the grading of the MRIs was a subjective grading, and although this is commonly performed in the ACL reconstruction literature, future studies with objective computational grading are needed. Finally, no arthroscopic validation of the findings was possible in the patients with stable examination. Prospective studies obtaining MRIs at regular intervals in the early rehabilitation phase (i.e., first 6 months) are therefore needed to assess healing and guide rehabilitation following arthroscopic primary ACL repair [23].

## 5. Conclusion

Postoperative MRI following arthroscopic primary ACL repair can be used to assess rerupture of the repaired ligament with excellent sensitivity (75%), specificity (90%), and accuracy (86%). Furthermore, it was noted that ligaments were often hyperintense in the first postoperative year, isointense between one and two years postoperatively, and hypointense and similar to the PCL after more than two years postoperatively.

## Figures and Tables

**Table 1:** Method of grading graft maturity in the ACL reconstruction literature.

MRI Appearance	Howell et al. [40]	Kanamiya et al. [41]	Figeroa et al. [39]
Part of graft evaluated	Complete graft	>50% of graft	>50% of graft
Reference	PCL	GM	SMT

Hypointense	Similar	< intense	< intense
Isointense	>50% similar	Similar	Similar
Hyperintense	<50% similar	> intense	> intense

ACL, anterior cruciate ligament; PCL, posterior cruciate ligament; GM, gastrocnemius muscle; SMT, semimembranosus tendon.

**Table 2:** Baseline characteristics of all patients in this study cohort and the reasons for the postoperative MRI.

*Baseline characteristics*	
Number of patients (MRIs)	36 (37)
Male patients	20 (57%)
Right side	21 (60%)
Age (years; mean ± SD (range))	30 ± 12 (range 14–57)
BMI (kg/m^2^; mean ± SD (range))	25 ± 4 (range 18–35)
Time from surgery to MRI (years; mean ± SD (range))	1.5 ± 1.1 (range 0.1–4.9)

*Reasons for postoperative MRI*	
Knee pain without trauma (n (%))	12 (32%)
Trauma with high suspicion for ACL re-injury (n (%))	9 (24%)
Evaluate ligament healing (n (%))	8 (22%)
Trauma with low suspicion for ACL re-injury (n (%))	4 (11%)
Superficial tenderness over suture anchor (n (%))	2 (5%)
Evaluate meniscal (root) repair (n (%))	2 (5%)

*Postoperative MRI*	
Unstable clinic examination (re-injury)	8 (22%)
Stable clinical examination (no re-injury)	29 (78%)
Within 1 year after primary repair	11 (38%)
Between 1 and 2 years after primary repair	10 (34%)
More than 2 years after primary repair	8 (28%)

MRI, magnetic resonance imaging; SD, standard deviation; BMI, body mass index; n, number.

**Table 3:** Assessment of intact ligament by the blinded radiologist.

		Clinical Stability on PE		
		Torn	Intact	Total
MRI	Torn	6	3	9
	Intact	2	26	28
		8	29	37

Sensitivity: 75%; specificity: 90%; positive predictive value: 67%; negative predictive value: 93%; accuracy: 86%; MRI, magnetic resonance imaging; PE, physical examination (consisting of Lachman, pivot, and anterior drawer examination).

## Data Availability

The data used to support the findings of this study are available from the corresponding author upon request.
